# Optimizing inhalation therapy in the aspect of peak inhalation flow rate in patients with chronic obstructive pulmonary disease or asthma

**DOI:** 10.1186/s12890-021-01674-5

**Published:** 2021-09-24

**Authors:** Jian-lan Hua, Xiao-fen Ye, Chun-ling Du, Ning Xie, Jie-qing Zhang, Man Li, Jing Zhang

**Affiliations:** 1 Department of Pulmonary and Critical Care Medicine, Zhongshan Hospital, Shanghai Medical College, Fudan University, 180 Feng Lin Road, Shanghai, 200032 People’s Republic of China; 2 Department of Pharmacy, Zhongshan Hospital, Shanghai Medical College, Fudan University, Shanghai, China; 3 Department of Pulmonary, Qingpu Branch of Zhongshan Hospital Affiliated to Fudan University, Shanghai, China; 4 Department of Pharmacy, Qingpu Branch of Zhongshan Hospital Affiliated to Fudan University, Shanghai, China

**Keywords:** Chronic obstructive pulmonary disease, Asthma, Dry powder inhalers, Peak inhalation flow rate, Technique training

## Abstract

**Background:**

Pressurized metered dose inhalers (pMDIs) and dry powder inhalers (DPIs) are commonly used drug-delivering devices for patients with chronic airway diseases. Appropriate peak inhalation flow rate (PIFR) and inhaler technique is essential for effective therapy. We aimed at optimizing inhalation therapy through the analysis of PIFRs in patients with chronic obstructive pulmonary disease (COPD) or asthma as well as the effect of technique training using In-Check DIAL® to help patients to achieve their optimal inspiratory flow rates.

**Methods:**

The study continuously enrolled patients who were diagnosed as COPD or asthma from respiratory clinics. PIFRs were described and analyzed between the newly-diagnosed and follow-up patients, and the stable and acute exacerbation patients, respectively. Every participant was trained inhaler technique using In-Check DIAL®. PIFRs before and after training was compared by self-control analysis.

**Results:**

Among a total of 209 patients, the average age was 56.9 years. For DPIs users, 10.8% patients had a PIFR < 30 L/min and 44.1% patients had a PIFR ≥ 60 L/min before technique training. After technique training, scarcely patient (1.5%) had a PIFR < 30 L/min, and 60.5% patients had a PIFR ≥ 60 L/min. The patient’s average PIFR increased by 5.6L/min after training. The increase in PIFR before and after training was significant (*p* < 0.001) for most patients, but no significant variation was found in patients with acute exacerbation (*p* = 0.822).

**Conclusions:**

A considerable number of patients with COPD or asthma were not able to achieve the minimum or optimal PIFR for DPIs. Inhaler training can increase patients’ PIFRs and improve their ability to use DPIs.

*Trail registration* The study has registered in chictr.org.cn (ChiCTR1900024707) and been approved by the Ethics Committee of Zhongshan Hospital of Fudan University (B2019-142).

## Introduction

Chronic respiratory diseases, especially Chronic obstructive pulmonary disease (COPD) and asthma, are common diseases worldwide with leading mortality and morbidity. In China, the prevalence of COPD in patients over 40 years old was 8.2–13.7% [1, 2]. COPD has accounted for 1.6% of all hospital admissions and ranked as the fourth leading cause of death in urban areas and the third leading cause of death in rural areas worldwide [3]. COPD has been a heavy burden for China, with a direct medical cost of $72 to $3565 per capita per year accounting for 40% of the average family's total income [4]. In the recent epidemiological studies, the overall prevalence of asthma in China ranged from 1.2 to 5.8%, while 4.2% among adults [5, 6].

Inhalation therapies, including inhaled corticosteroid (ICS), long-acting β2 agonists (LABA) and long-acting muscarinic antagonists (LAMA), play an important role for the treatment and management of both COPD and asthma [7]. Inhalers typically used for inhalation therapy are sorted into three types based on their respective technical characteristics and particle properties: pressurized metered dose inhaler (pMDI), dry powder inhalers (DPIs), soft mist inhalers (SMI). pMDIs do not require the patients’ peak inhalation flow rate (PIFR) to reach a certain value, but drug delivery using pMDIs is highly dependent on the patient’s inhaler technique [8]. Failure to coordinate or synchronize actuation with inhalation leading to suboptimal lung deposition are commonplace reported in previous studies [9]. In comparison, DPIs are essentially breath-actuated and easier to use correctly than pMDIs, but demand patients to generate a sufficient inspiratory flow to release the powder and break up the powder packets into respirable particles (less than 5 μm in diameter) [10].

Recently, PIFR has been believed as a measure to assess patients' capacity to use DPIs [11]. DPIs approved for treatment of COPD and Asthma include the HandiHaler, Turbuhaler, Aerolizer, Accuhaler/Diskus, Breezhaler, Genuair/Pressair, etc. The recommended technique for patients when using DPIs is ‘a fast and hard inhalation’. Due to the difference in the internal resistance of devices, the level of resistance that the patient needs to overcome when using different DPIs varies. For example, using DPIs with high resistance like Turbuhaler and HandiHaler require more inspiratory effort than using those with low resistance like Breezhaler. Patients using DPIs need to achieve a minimum inhalation rate for the effective clinical response or ideally an optimal rate for the best response. Given previous studies, it is generally considered that PIFR less than 30 L/min is insufficient for the use of DPIs [12]. PIFR of at least 60L/min achieved by patients can bring about optimal drug delivery through DPIs [13]. Unlike DPIs, the technical essential for patients when using pMDI is ‘a slow and deep inhalation’, which requires that the patient's PIFR should be less than 90 L/min [14]. However, observational studies demonstrate that 19% of patients with stable COPD or asthma [15] and 32% to 47% of in-patients prior to discharge after recovering from exacerbation suffered a suboptimal PIFR (less than 60L/min) [16]. Moreover, the PIFRs of 12% of elder Turbuhaler users were even lower than the minimum effective rate (30 L/min) [17]. If patients' inspiratory flow rate does not match DPIs, the insufficient PIFR associating with the dose of inhaled drugs poorly deposited in lung will result in unsatisfied efficacy and potentially poor prognosis [18, 19]. Overall, PIFR is an important consideration for physicians to choose an appropriate inhaler for patients.

Appropriate technique for the usage of inhalers is quite important for the efficacy of inhalation therapy that improper technique is significantly associated with uncontrolled symptoms and increased exacerbation rate [20]. For patients using DPIs and pMDIs, the most critical and common technique errors are inappropriate inspiratory maneuver and poorly synchronized hand actuation with inhalation, respectively [21]. Several reports have revealed that up to 70%-80% of patients made at least 1 inhalation technique error when using DPIs, and 86%-87% of patients when using pMDIs [22, 23]. Especially, patients using Turbuhaler are most likely to make mistakes [24]. Therefore, enhancing patients’ inhaler technique through teaching and training may contribute to improving prognosis and decreasing medical expenditure.

In the current study, we aimed to investigate the PIFRs of patients with COPD or asthma, factors that affect PIFRs and the effect of inhaler technique training on optimizing patients’ PIFRs before inhalation therapy. Through this study, the optimized inhalation therapy based on PIFR should be guided both in selection of the most acceptable inhaler for patients and in training to improve inhaler technique.

## Methods

### Study design and recruitment

We conducted a prospective, self-control, single-center study at Respiratory Clinic in Zhongshan Hospital of Fudan University, Shanghai, China. All participants have signed an informed consent before being recruited. The study has registered in chictr.org.cn (ChiCTR1900024707) and been approved by the Ethics Committee of Zhongshan Hospital of Fudan University (B2019-142).

This study continuously enrolled patients who were diagnosed as COPD or asthma and prescribed inhalers attending Zhongshan Hospital of Fudan University from June 2020 to September 2020. Patients who were diagnosed with COPD or asthma were required to meet the diagnostic criteria defined by *Global Strategy for the Diagnosis, Management, and Prevention of Chronic Obstructive Lung Disease(GOLD) 2019 Report* [7] or *Global Strategy for Asthma Management and Prevention (GINA 2019 update)* [25] respectively, including medical history, symptoms and pulmonary function tests. For every patient enrolled in the study, a clear pulmonary function test result supporting the diagnosis was necessary (for COPD, FEV_1_/FVC < 0.7 post bronchodilator [7]; for asthma, an increase or decrease in FEV_1_ of > 12% and 200 ml from baseline, or a change in PEF of at least 20% [25])*.* Likewise, an exacerbation of asthma represents a change in symptoms and lung function from the patient's usual status in the light of GINA [25], and an exacerbation of COPD is defined as an acute worsening of respiratory symptoms that results in additional therapy in the light of GOLD report [7].

Exclusion criteria include the following: (1) the patient himself/herself did not participate in the consultation; (2) the patient was concomitant with interstitial lung disease, bronchiectasis, pulmonary embolism, and other lung diseases; (3) the patient suffered from cognitive impairment or not cooperating with the study due to poor mental state; (4) the patient did not agree to sign the informed consent.

### Data collection

Demographic and clinical characteristics of participants with spirometry-diagnosed COPD or asthma were collected by researchers upon enrollment including gender, age, history of smoking, number of exacerbations in the past year, previous use of inhalation therapy, the severity and control of asthma, and GOLD severity classification (only for COPD patients). Meanwhile, participants were asked to fill out COPD Assessment Test (CAT) (only for patients with COPD) and modified Medical Research Council Dyspnea Scale (mMRC) (for all patients). The following data were also recorded: forced expiratory volume in 1 s (FEV_1_), FEV_1_/predicted FEV_1_ (FEV_1_%), forced expiratory volume in 1 s/forced vital capacity (FEV_1_/FVC), peak expiratory flow (PEF), inspiratory capacity (IC), residual volume/total lung capacity (RV/TLC).

The researchers measured PIFR using In-Check® DIAL (Clement Clarke International, Harlow, UK and Alliance Tech Medical). Researches orally taught patients about inhaler techniques and the inhalation maneuver was trained by the device, and PIFR was measured again. All data collection was completed on the day of enrollment.

### Peak inhalation flow rate measurement at different resistance

In the study, patients' PIFR was measured by In-Check DIAL®, which is designed to measure inspiratory flow and provides an adjustable dial with different sized openings to simulate resistances of various inhalers. It is accurate up to ± 10% or 10 L/min and is a low-range inspiratory flow metre (15–120 L/min) with options for resistance ranging from high to low. The In-Check DIAL® contains six levels of resistance groups for both pMDIs and DPIs classified as "pMDI"(at resistance of zero), “low” (simulating Breezhaler®), “medium low” (simulating Accuhaler®, Diskhaler®, Ellipta®), “medium” (simulating Spiromax®, GenuAir®, Clickhaler®, Turbohaler®), “medium high” (simulating Turbohaler®, Easyhaler®, Twisthaler®, NEXThaler®), and “high” (simulating Handihaler®, Easyhaler®M). PIFRs for pMDI devices were measured at no resistance and indicated as PIFR_0_. PIFRs at the resistance of corresponding levels simulating the prescribed DPI inhalers were measured before and after inhaler technique training, which were indicated as before-training PIFR (PIFR_BT_) and after-training PIFR (PIFR_AT_), respectively. Specifically, for DPI users, PIFRs at the resistance of prescribed devices were measured for 3 times and the best optimal one was recorded as PIFR_BT_. Then the patients were trained to use the inhaler (as described in Inhaler technique training by In-Check DIAL® below). After training, the best optimal value of 3 repeated measurements were recorded as PIFR_AT_..

All PIFR measurements are performed in a separate, private, and quiet consulting room on the day of enrollment. For patients with the exacerbation of asthma or COPD, their PIFRs were measured within 1–3 days of the onset of the exacerbation..

### Inhaler technique training by In-Check DIAL®

The In-Check DIAL® can serve as a training device for inhalation muscles and contribute to improving patients’ skills to use inhalers. In this study, a total of 3 researchers participated in inhaler technique training. Before the beginning of the study, we had conducted uniform training and testing for these researchers on educational skills about usage of all types of inhalers and In-Check Dial®. Meanwhile, we had prepared standardized inhaler demonstration videos and manuals for patients. After patients were prescribed different inhalers, the researchers taught and trained their usage technique of respective inhalers. Researcher introduced the characteristics of the inhaler, demonstrated method of application and emphasized technical points to patients in the form of instructional videos and orally. Then patients were asked to train the inhaler technique using In-Check DIAL® with the resistance simulating the corresponding inhaler. Investigators were supposed to correct their technical errors during training until the patients used the inhaler correctly and reached an optimal PIFR as much as possible.

### Statistical analysis

Descriptive statistics were used to characterize the demographic and clinical characteristics of patients and the distribution of PIFR in all patients and subgroups (continuous variables including value of PIFR_0_, PIFR_BT_ and PIFR_AT_ are described as mean ± standard deviation, while categorical variables including the number of patients whose PIFR was less than 30L/min, 30 L/min to 60L/min or greater than 60L/min were described as frequency and percentage). We divided all patients to 2 subgroups in 2 different ways, namely the newly-diagnosed group/follow-up group (ND group/FU group), and the stable group/acute exacerbation group (stable group/AE group). The values of PIFR_0_, PIFR_BT_ and PIFR_AT_ between subgroups were compared using Mann–Whitney U test or independent-sample t test. The comparison of self-control group (PIFR_BT_ vs. PIFR_AT_) was performed by paired-samples t test (for the value of PIFR) or McNemar's test (for the categorical variables of PIFR). Due to the range limitation of In-Check Dial®, the PIFR_0_ we measured did not obey the normal distribution. Considering the related variables analyzed include categorical variables such as sex, the correlation of PIFR_0_ with lung function index, CAT score and mMRC score were estimated using Spearman rank correlation analysis to ensure the consistency of the results The general significance level was set to 0.05. All statistical analyses were conducted using IBM SPSS Statistics V.22 (IBM Corporation, Armonk, NY, USA).

## Results

### Demographic characteristics of participants

A total of 209 patients who met all inclusion criteria and with no exclusion items were continuously enrolled at the period of patient recruitment in the study, and none of the invited patients refused to participate. The demographic characteristics of the participants were presented in Table 1 (the patients with both asthma and COPD were categorized into COPD). Among all participants, there were 126 males (60.3%). The average age of all participants was 56.9 ± 17.6(mean ± SD) years, and 93 patients (45.6%) had a history of smoking.

**Table 1:** Demographic characteristics of participants

	Asthma (n = 93)	COPD (n = 116)	Total (n = 209)
Sex, male (%)	37 (39.8)	89 (76.7)	126 (60.3)
Age (years)	44.9 ± 15.4	66.6 ± 12.7	56.9 ± 17.6
**Smoking status (%)**			
Yes (including current and former smokers)	19 (21.1)	74 (64.9)	93 (45.6)
Current	14 (15.5)	27 (23.7)	41 (20.1)
Former	5 (5.6)	47 (41.2)	52 (25.5)
Never	66 (73.3)	38 (33.3)	104 (51.0)
Secondhand	5 (5.6)	2 (1.8)	7 (3.4)

Data are shown as means ± standard deviation or number (%) patients

COPD, chronic obstructive pulmonary disease

### Clinical characteristics of participants

The clinical characteristics of all participants including 93 patients with asthma and 116 patients with COPD are shown in Table 2. Among all patients, 77 patients were newly diagnosed as COPD or asthma who were prescribed inhalation therapy for the first time (ND group), 132 patients were followed up (FU group); 34 patients with the exacerbation of COPD or asthma and 174 patients with stable COPD or asthma.

**Table 2:** Clinical characteristics of participants

	Asthma (n = 93)	COPD (n = 116)	Total (n = 209)
**Stable /AE status**			
AE (%)	10 (10.8)	24 (20.9)	34 (16.3)
Stable (%)	83 (89.2)	91 (79.1)	174 (83.7)
**Newly diagnosed/follow-up patients**			
Newly-diagnosed (%)	41 (44.1)	36 (31.0)	77 (36.8)
Follow-up (%)	52 (55.9)	80 (69.0)	132 (63.2)
**Number of acute exacerbations in the past year**			
0 (%)	75 (80.6)	82 (70.7)	157 (75.1)
1 (%)	10 (10.8)	18 (15.5)	28 (13.4)
2 (%)	3 (3.2)	7 (6.0)	10 (4.8)
≥ 3 (%)	5 (5.4)	9 (7.8)	14 (6.7)
**Pulmonary function**			
FEV_1_ (L)**,** mean ± SD	2.3 ± 0.8	1.4 ± 0.6	1.8 ± 0.8
FEV_1_, %predicted**,** mean ± SD	81.5 ± 21.4	51.7 ± 22.0	65.5 ± 26.3
FEV_1_/FVC, mean ± SD	74.4 ± 10.9	55.7 ± 11.6	64.3 ± 14.6
PEF (L), mean ± SD	5.9 ± 1.9	4.0 ± 1.8	4.9 ± 2.1
IC (L), mean ± SD	2.3 ± 0.6	2.0 ± 0.7	2.1 ± 0.7
RV/TLC, mean ± SD	43.1 ± 9.0	51.0 ± 9.8	47.3 ± 10.2
**GOLD**			
I (%)	/	12 (14.5)	/
II (%)	/	23 (27.7)	/
III (%)	/	36 (43.4)	/
IV (%)	/	12 (14.5)	/
**Severity of asthma**			
Mild and moderate asthma (%)	86 (92.5)	/	/
Severe asthma (%)	7 (7.5)	/	/
**Control of asthma**			
Well and partly controlled (%)	83 (89.2)	/	/
Uncontrolled (%)	10 (10.8)	/	/
CAT, mean ± SD	/	10.5 ± 6.5	/
mMRC, mean ± SD	0.4 ± 0.7	1.2 ± 1.0	0.8 ± 0.9

Data are shown as means ± standard deviation or number (%) patients

COPD, chronic obstructive pulmonary disease; AE, acute exacerbation; FEV_1_, forced expiratory volume in 1 s; FEV_1_%, FEV_1_/predicted FEV_1_; FEV_1_/FVC, forced expiratory volume in 1 s/forced vital capacity; PEF, peak expiratory flow; IC, inspiratory capacity; RV/TLC, residual volume/total lung capacity; GOLD, Global initiative for Chronic Obstructive Lung Disease; CAT, COPD Assessment Test; mMRC, Medical Research Council Dyspnea Scale

### Measurements of PIFRs

Table 3 showed the distribution of PIFR in all patients and patients with different clinical conditions. The PIFR in Table 3 includes PIFR_0_ of all patients, but PIFR_BT_ and PIFR_AT_ only for patients using DPIs. 61 participants (28.9%) have used 2 or more inhalers. The average PIFR_0_ of all patients was 101.7 ± 24.7L/min, among which the average PIFR_0_ was 103.0 ± 24.6L/min for patients with asthma and 100.7 ± 24.9L/min for patients with COPD, respectively (*p* = 0.445, U = 5705.0). The average PIFR_0_ was 94.2 ± 28.2 L/min for ND group and 106.1 ± 21.4L/min for FU group, respectively (*p* < 0.001, U = 6522.0); the average PIFR_0_ was 96.9 ± 26.3L/min for AE group and 102.9 ± 24.3L/min for stable group, respectively (*p* = 0.143, U = 2517.5).

**Table 3:** Distribution of PIFR

	Patients using 2 or more inhalers (%)	Patients using 2 or more DPIs (%)	PIFR_0_, L/min	PIFR_BT_, L/min	PIFR_AT_, L/min
**All patients**	61 (28.9)	27 (12.9)	118.0 (90.0,120.0)	55.4 ± 21.1	61.0 ± 18.8
**Asthma/COPD group**					
Asthma group	14 (15.1)	5 (5.4)	120.0 (90.0,120.0)	56.6 ± 20.6	62.9 ± 17.4
COPD group	47 (40.2)	22 (18.8)	115.0 (85.0,120.0)	54.7 ± 21.5	60.0 ± 17.8
*P* value (MD, 95%CI)	/	/	0.445	0.541 (MD = 1.9, 95% CI [−4.3, 8.1])	0.278 ((MD = 3.2, 95% CI [−2.6, 8.9])
**ND/FU group**					
ND group	10 (13.0)	5 (6.4)	105.0 (70.0, 120.0)	47.8 ± 18.0	55.9 ± 16.1
FU group	51 (38.3)	22 (16.6)	120.0 (95.0, 120.0)	60.0 ± 21.6	64.0 ± 17.9
*p* value	/	/	0.000	0.000 (MD = 12.1, 95%CI [6.2,18,1])	0.002 (MD = 8.9, 95%CI [3.3,14.5])
**AE/stable group**					
AE group	19 (54.3)	8 (22.9)	120.0 (90.0, 120.0)	51.3 ± 18.5	51.8 ± 11.4
Stable group	42 (24.1)	19 (10.9)	110.0 (77.5, 120.0)	56.5 ± 21.5	63.3 ± 17.9
*p* value	/	/	0.143	0.192 (MD = 5.2, 95% CI [−2.6,13.0])	0.000 (MD = 13.0, 95% CI [−5.9, 20.1])

Patients using 2 or more inhalers/DPIs are shown as number (%) patients. Data are shown as means ± standard deviation or median (25% quartile, 75%quartile). The PIFR data in Table 3 includes PIFR_0_ of all patients, and PIFR_BT_ and PIFR_AT_ only for patients using DPIs

CI, confidence interval; COPD, chronic obstructive pulmonary disease; AE, acute exacerbation; ND, newly-diagnosed; FU, follow-up; PIFR_0_, peak inhalation flow rate measured at resistant of “pMDI”; PIFR_BT_, peak inhalation flow rate measured before-training; PIFR_AT_, peak inhalation flow rate measured after training; MD, mean difference

**Fig. 1:**
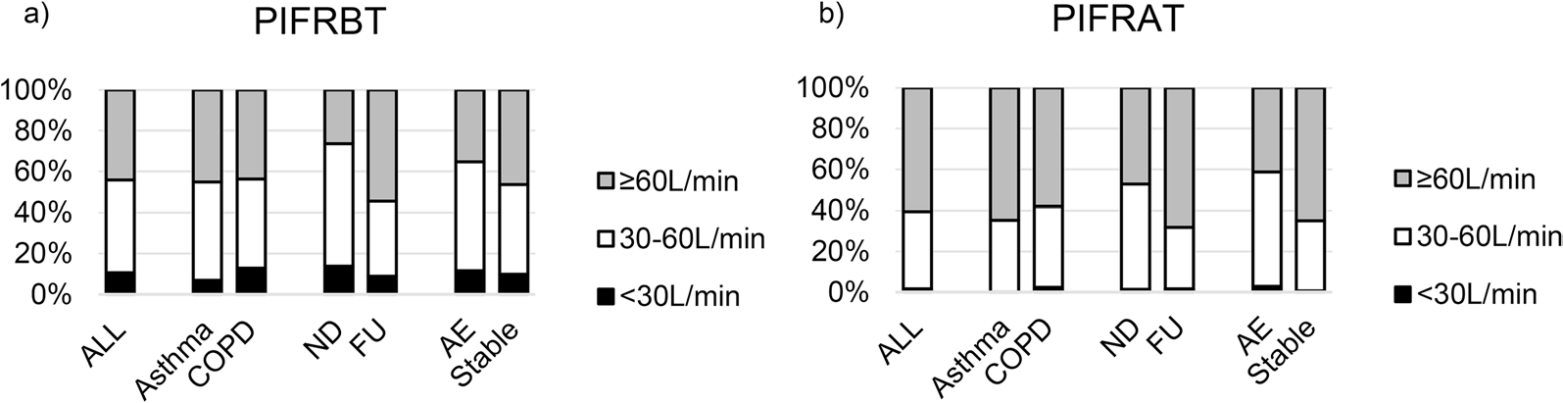
Percentage of different PIFR_BT_ and PIFR_AT_. Figure is percent stacked column charts of the PIFR distribution for patients using DPIs before and after technique training. **a** There were 21 (10.8%) patients with a PIFR_BT_ < 30L/min and 86 (44.1%) patients with a PIFR_BT_ ≥ 60L/min among all patients. The population and proportions of patients with a PIFR_BT_ < 30L/min in ND/FU group were 10 (13.9%)/11(8.9%). The population and proportions of patients with a PIFR_BT_ ≥ 60L/min in ND/FU group were 19 (26.4%)/67 (54.5%). **b** There were 3 (1.5%) patients with a PIFR_AT_ < 30L/min and 118 (60.5%) patients with a PIFR_AT_ ≥ 60L/min among all patients. COPD, chronic obstructive pulmonary disease; AE, acute exacerbation; ND, newly-diagnosed; FU, follow-up; PIFR_BT_, peak inhalation flow rate measured before-training; PIFR_AT_, peak inhalation flow rate measured after training

27 participants (12.9%) have used 2 or more DPIs. As shown in Fig. 1, 21 patients (10.8%) had a PIFR_BT_ less than 30 L/min, and 86 patients (44.1%) had a PIFR_BT_ greater than or equal to 60 L/min before technique training. 13.9%/26.4% newly-diagnosed patients had a PIFR_BT_ < 30 L/min/ ≥ 60 L/min; as contrasted, 8.9%/54.5% follow-up patients had a PIFR_BT_ < 30 L/min/ ≥ 60 L/min. Relatively, scarcely patient (only 3 patients, 1.5%) had a PIFR_AT_ less than 30 L/min, and a rather high 118 patients (60.5%) had a PIFR_AT_ greater than or equal to 60 L/min after technique training. The group of AE contained a total of 34 patients, consisting of 10 patients with asthma and 24 with COPD. We did not find a significant difference in PIFR_BT_ (*p* = 0.350, 95% CI [−5.7.22.4]) or in PIFR_AT_ (*p* = 0.784, 95% CI [−7.0, 11.7]) between the patients with asthma exacerbation and COPD exacerbation. Additionally, PIFR_AT_ of the AE group was significantly lower than that of the stable group (*p* < 0.001, MD = 13.0, 95% CI [−5.9, 20.1]). Even after training, 58.8% of AE patients still failed to achieve optimal PIFR.

### Optimal PIFRs rates improved after technique training

**Fig. 2:**
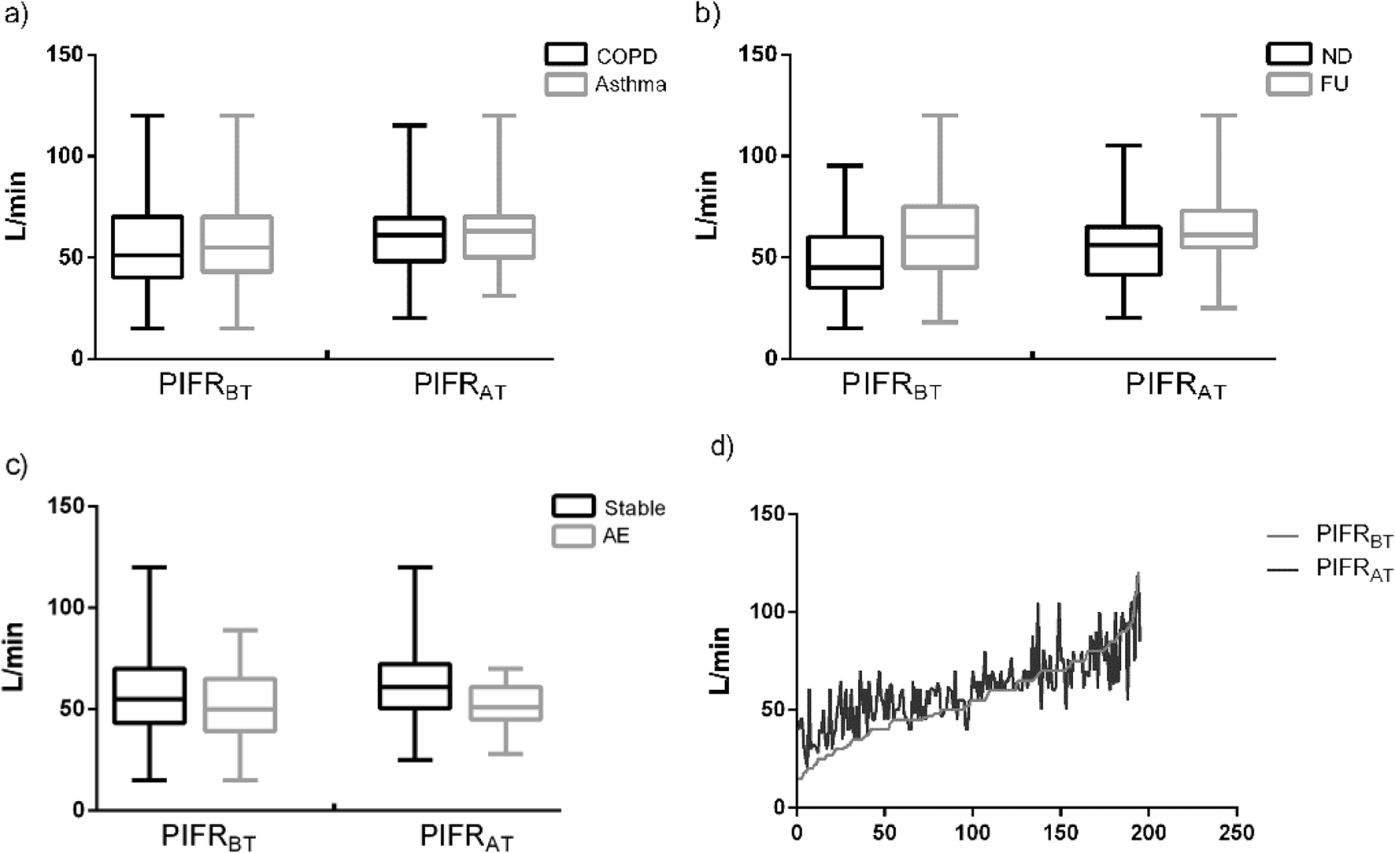
Distribution and variation trend of PIFR in COPD/Asthma group, ND/FU group, AE/Stable group and all patients. Figure describes the distribution and changes of PIFR_BT_ and PIFR_AT_ for patients using DPIs in COPD/Asthma group, ND/FU group, AE/Stable group and all patients. **a** is a box diagram showing the improvement from PIFR_BT_ to PIFR_AT_ in COPD/Asthma group. **b** is a box diagram showing the improvement from PIFR_BT_ to PIFR_AT_ in ND/FU group. **c** is a box diagram showing the improvement from PIFR_BT_ to PIFR_AT_ in AE/Stable group. **d** is a line chart for PIFR_BT_ and PIFR_AT_ in all patients, which the two values corresponding to each abscissa value are PIFR_BT_ and PIFR_AT_ of the same patient. When the patient's PIFR was relatively low, training increases the PIFR value more obviously. COPD, chronic obstructive pulmonary disease; AE, acute exacerbation; ND, newly-diagnosed; FU, follow-up; PIFR_BT_, peak inhalation flow rate measured before-training; PIFR_AT_, peak inhalation flow rate measured after training

In a total of 209 subjects, 166 (79.4%) subjects were using DPIs. Figure 2 showed the distribution and training-associated changes of PIFRs in patients of different clinical conditions using DPIs. The average PIFR_BT_ and PIFR_AT_ in all patients was 55.4 ± 21.1 L/min and 61.0 ± 18.8 L/min respectively (see Table 3) (*p* < 0.001, MD = 5.2, 95% CI [3.8, 7.4]). The similar increased PIFRs after training were found in patients with both COPD and asthma, new-diagnosed and follow-up patients (p all < 0.001, COPD group: MD = 5.3, 95% CI [3.0, 7.6]; Asthma group: MD = 6.3, 95% CI [3.2, 9.3]; ND group: MD = 8.2, 95% CI [5.8, 10.5]; FU group: MD = 4.1, 95% CI [1.6, 6.67]). However, the patients' PIFR_AT_ showed a significant improvement over PIFR_BT_ in Stable group but there was no significant difference between PIFR_AT_ and PIFR_BT_ in AE group (Stable group: *p* < 0.001, MD = 6.8, 95% CI [4.8, 8.8]; AE group: *p* = 0.822, MD = 0.5, 95% CI [−3.8 ,4.7]). Meanwhile, McNemar's test demonstrated that the percentages of patients reaching minimum PIFR (30 L/min) and optimal PIFR (60 L/min) after technique training were both significantly improved (*p* < 0.001, χ^2^ = 16.1, 25.3). Furthermore, the similar improvement in the percentages of patients reaching minimum PIFR and optimal PIFR after training were found in patients with both COPD and asthma, new-diagnosed and follow-up patients (*p* all < 0.005, except for *p* value of improvement in minimum PIFR achieving by patients with asthma was 0.063). It should be noted that the significant improvement in PIFRs after training was not found in AE group, either in asthma exacerbators (*p* = 0.250, χ^2^ = 1.3) or COPD exacerbators (*p* = 0.625, χ^2^ = 0.3).

In our study, 13(16.3%) of the patients who used pMDIs (N = 80) had a PIFR greater than 90 L/min before training, among them 7 (46.2%) were COPD patients and 6 (53.8%) were asthmatic patients. Most of them 12 (92.3%) had stable diseases. Compared with subjects with PIFR less than 90L/min, they had similar lung function (FEV_1_%: 59.7 ± 30.7 vs 58.8 ± 27.2, *p* = 0.925, 95% CI [−20.1, 18.3]) and symptoms (mMRC: 0.8 ± 1.2 vs 1.2 ± 1.0, *p* = 0.225, 95% CI [−0.3, 1.1]). Although training has improved the patients’ inhaler technique of pMDI to some extent, but it was not statistically significant (*p* = 0.065, χ^2^ = 3.3). It is worth noting that there were still 7.5% subjects using pMDIs with a PIFR greater than 90L/min after training.

### Clinical factors that effect PIFRs

As shown in Table 4, Spearman test demonstrated that PIFR_0_ was not significantly relative to sex, BMI (body mass index), FEV_1_%, FEV_1_/FVC, RV/TLC and CAT score, but was weakly relative to FEV_1_, PEF, IC, and mMRC score. Among them, PIFR_0_ was positively correlated with FEV_1_, PEF, and IC (*p* values are 0.013, 0.008, 0.001, and r values are 0.199, 0.218, and 0.284, respectively). On the contrary, PIFR_0_ showed a negative correlation with mMRC score (*p* = 0.005, r = -0.200).

**Table 4:** Relativity between PIFR_0_ and clinical indicators

Clinical indicators	*p* value	R (95% CI)
Sex	0.061	−0.130 (−0.261, 0.016)
BMI	0.312	0.071 (−0.076, 0.248)
FEV_1_	0.013*	0.199 (0.072, 0.351)
FEV_1_, %predicted	0.900	−0.010 (−0.172, 0.149)
FEV_1_/FVC	0.948	0.005 (−0.149, 0.169)
PEF	0.008*	0.218 (0.097, 0.356)
IC	0.001*	0.284 (0.097, 0.422)
RV/TLC	0.255	−0.098 (−0.296, 0.084)
CAT	0.632	−0.047 (−0.230, 0.162)
mMRC	0.005*	−0.200 (−0.346, −0.044)

Except for FEV_1_ (%predicted), which is the percentage of predicted value, the other pulmonary function parameters are absolute values

*r,* Spearman rank correlation coefficient; BMI, body mass index; FEV_1_, forced expiratory volume in 1 s; FEV_1_%, FEV_1_/predicted FEV_1_; FEV_1_/FVC, forced expiratory volume in 1 s/forced vital capacity; PEF, peak expiratory flow; IC, inspiratory capacity; RV/TLC, residual volume/total lung capacity; CAT, COPD Assessment Test; mMRC, Medical Research Council Dyspnea Scale.

*p value < 0.05

## Discussion

Different inhalers have different requirements for the patient's inspiratory flow rate and technique [26]. Although the types of inhalers available to doctors and patients are increasing day by day, there are still many patients whose PIFR does not match the requirements of the inhaler or the inhaler technique is not qualified, resulting in suboptimal efficacy of inhalation therapy [27, 28]. In this prospective, self-control, single-center study, the number of patients with very severe COPD (GOLD IV, defined as FEV_1_% < 30%) is relatively small (12 patients, 14.5%) since the patients we enrolled were all outpatients. Though after training, 39.5% of patients were not yet able to achieve the optimal PIFR (60L/min), which showed that there were still quite a few patients with a suboptimal inspiratory flow rate even in patients with mild, moderate or severe COPD (GOLD I–III).

In this study, in addition to routine instructional video and oral guidance by physicians, we additionally emphasized the application of In-Check DIAL® in technique training. Our study has shown that 10.8% of patients using DPI could not achieve the minimum PIFR (30L/min) and 55.9% of patients could not achieve the optimal PIFR (60L/min) before technical training. After training, almost no patients had a PIFR less than 30L/min, and the number of patients who did not reach the optimal flow rate was significantly reduced, which revealed that technique training helped to significantly increased both the value of PIFR and the proportion of patients that reached the minimum and optimal flow rate. Our attempt to train patients with In-Check DIAL® truly contributed to improving PIFR of patients with COPD or asthma. Moreover, the improvement of PIFR in COPD patients before and after training was more significant than that of asthmatic patients. Therefore, we considered that COPD patients need more training, probably because asthma patients were younger and stronger in learning ability than COPD patients.

We have investigated the distribution of PIFRs in patients with different clinical conditions in this study. By comparing PIFR (including PIFR_0_, PIFR_AT_ and PIFR_BT_) in new-diagnosed (indicating no effective treatment had ever be used) vs. follow-up patients and patients in exacerbation vs. stable stage, we found that PIFR of ND/FU group or AE/Stable group showed a significant difference. It meant that compared with follow-up patients, newly-diagnosed patients who were given inhalation therapy for the first time benefited more from inhaler technique training in improving the ability of using DPIs; and compared with AE patients, patients in the stable phase benefited more in improving PIFR.

From the distribution of PIFR_AT_ and PIFR_BT_, it is not difficult to find that among all the groups before teaching, ND group had the largest proportion of PIFR_BT_ less than 30 L/min (13.9%) and the smallest proportion of PIFR_BT_ greater than or equal to 60 L/min (26.4%), but FU group had the largest proportion of PIFR_BT_ greater than or equal to 60 L/min (54.5%). These data suggest that newly diagnosed patients should strengthen knowledge education and inhaler technique training, and pMDIs may be prescribed for some patients with suboptimal PIFR (DPIs can be prescribed after improvement).

Among all the groups after teaching, AE group had the least proportion of PIFR_AT_ greater than or equal to 60 L/min (41.2%), which is significantly lower than that of the stable group (68.3%). In the AE group, the improvement in PIFR before and after training (P > 0.050) did not reach statistical significance, indicating that many AE patients had poor inhalation ability and the benefits of training for AE patients were limited. It can be found that when the influence of technical factors on PIFR is excluded, AE patients had a higher suboptimal rate and a worse ability to use DPIs than stable patients, but physicians kept giving DPIs to them (AE patients used 1.00 DPIs per capita). Previous researches have shown similar conclusions that acute exacerbations were associated with decrease in PIFR [27, 29]. For example, Palen et al. found that 50% of patients with an exacerbation of asthma or COPD were unable to generate optimal PIFR using Turbuhaler (compared with 5% of those without AE) [30]. All these data have proved that more AE patients were suitable to be prescribed SMIs or pMDIs rather than DPIs.

According to the study by Duarte et al., lung function measurements demonstrated a significant lower FEV_1_, total lung capacity (TLC), inspiratory capacity (IC) and a significant greater RV/TLC in the suboptimal PIFR group compared to the optimal PIFR group [31]. By contrast, Ghosh et al. demonstrated that the only factor found to be consistently associated with a lower PIFR was female gender, but there was a lack of consistent correlation between PIFR and FEV_1_ or FEV_1_% predicted or FVC or CAT score [26, 32]. In fact, the factors affecting PIFR are still controversial. In this study, we found a weak positive correlation between PIFR of outpatients with FEV_1_, PEF and IC (r = 0.199, 0.218, and 0.284), and a weak negative correlation with mMRC score (r = -0.200). However, our attempt to construct a qualified prediction model for PIFR based on these factors was a failure. At this stage, it is still necessary to measure PIFR by In-Check DIAL® to guide inhaler choice.

In addition, a slow and deep inhalation is required when using pMDIs, and a PIFR greater than 90L/min is considered too fast. Some studies have proven the necessity to educate patients regarding correct pMDI technique [33, 34]. Our results showed that training reduced the PIFR value of patients, but it was not statistically significant, which implied that technique training had potential impact for patients to use pMDIs correctly in the aspect of optimal PIFR.

## Conclusions

In general, a considerable number of outpatients with COPD or asthma were not able to achieve the optimal PIFRs for using DPIs. Inhaler education including training using In-Check DIAL® played an important role in improving patients’ PIFRs and we recommend that all patients who are prescribed inhalers should have their abilities evaluated and techniques trained. PIFR was associated with patients’ FEV_1_, PEF, IC and mMRC, but the correlation was not strong enough to indicate optimal PIFR to use specific DPIs. Besides, patients with AECOPD or asthma attack generally had suboptimal PIFRs and should be prescribed DPIs with caution.

## Data Availability

The datasets generated and/or analysed during the current study are not publicly available due to policy requirements but are available from the corresponding author on reasonable request.

## Abbreviations

COPD: Chronic obstructive pulmonary disease
AE: Acute exacerbation
BMI: Body mass index
FEV_1_: Forced expiratory volume in 1 s
FEV_1_%: FEV_1_/predicted FEV_1_
FEV_1_/FVC: Forced expiratory volume in 1 s/forced vital capacity
PEF: Peak expiratory flow
IC: Inspiratory capacity
RV/TLC: Residual volume/total lung capacity
CAT: COPD Assessment Test
mMRC: Medical Research Council Dyspnea Scale
ND: Newly-diagnosed
FU: Follow-up
PIFR_0_: Peak inhalation flow rate measured at resistant of “pMDI”
PIFR_BT_: Peak inhalation flow rate measured before-training
PIFR_AT_: Peak inhalation flow rate measured after training
